# Interaction between gut microbiota and anesthesia: mechanism exploration and translation challenges focusing on the gut-brain-liver axis

**DOI:** 10.3389/fcimb.2025.1626585

**Published:** 2025-09-08

**Authors:** Ruolan Zhang, Li Li, Gaojie Yu, Yang Li, Kexi Wei, Liang Lin, Yifeng Ye

**Affiliations:** ^1^ Department of Anesthesiology, The First Affiliated Hospital of Xiamen University, Xiamen, China; ^2^ Xiamen Anesthesia Quality Control Center, Xiamen, China; ^3^ School of Medicine, Xiamen University, Xiamen, China; ^4^ Department of Anesthesiology, Zhangzhou Pinghe County Hospital, Zhangzhou, Fujian, China; ^5^ School of Clinical Medicine, Fujian Medical University, Fuzhou, Fujian, China

**Keywords:** gut microbiota, anesthesia, gut-brain-liver axis, drug metabolism, clinical translation

## Abstract

As a core participant in human metabolism, immunity, and neural regulation, the gut microbiota has been demonstrated to be closely related to anesthesia drug metabolism and perioperative complications in recent years. Via the bidirectional interaction between the gut-brain axis and gut-liver axis, the gut microbiota and its metabolites can regulate central nervous system inflammation, liver drug-metabolizing enzyme activity, and the clearance efficiency of anesthetic drugs. Moreover, anesthetic drugs can significantly reshape the gut microbiota structure by altering intestinal barrier function, inhibiting beneficial bacterial proliferation, or inducing bile acid metabolism disorders, thereby resulting in a vicious cycle of neuroinflammation and metabolic abnormalities. Microbiota-targeted intervention strategies have demonstrated potential in alleviating anesthesia-related complications in response to this interactive network; however, their clinical translation is still limited by incomplete understanding of the underlying mechanisms, individual heterogeneity, and safety challenges. In the future, it will be necessary to integrate multiomics technologies for analyzing the tripartite interaction network of microorganisms, hosts, and drugs, as well as for promoting standardized clinical research, in order to develop individualized anesthesia management plans based on gut microbiota regulation; these initiatives can result in improvements in perioperative safety and patient prognosis.

## Introduction to the gut microbiota

The intestine represents the core organ for digestion and absorption in the human body; additionally, it is also a key site for maintaining immune homeostasis and metabolic balance. The implementation of intestinal function highly relies on the complex microbial community that coexists within the intestine (known as the gut microbiota). The gut microbiota consists of diverse microorganisms, including bacteria, archaea, eukaryotes, viruses, and parasites, that inhabit the intestine (Bradley and Haran, 2024). The number of microorganisms in the intestine is enormous, and it is estimated that the ratio of bacteria alone to human cells is close to 1:1 (Sender et al., 2016). In addition, the gene count in the gut microbiota is about 100 times higher than that found in the human body (Gill et al., 2006).

Recent advancements in high-throughput and cost-effective sequencing techniques have significantly enhanced our capacity to explore the diversity of the gut microbiota. Metagenomic analysis of the DNA encoding 16S rRNA has revealed that the gut microbiota involves seven phyla, including *Firmicutes*, *Bacteroidetes*, *Proteobacteria*, *Actinobacteria*, *Verrucobacteria*, *Clostridium*, and *Cyanobacteria*, with *Firmicutes* and *Bacteroidetes* representing the dominant bacteria (Breuninger et al., 2021; Olofsson and Bäckhed, 2022). The spatial distribution of the gut microbiota is highly heterogeneous; specifically, beginning from the distal esophagus (approximately 10^1^ CFU/g) and extending to the colon (10^12^ CFU/g), the microbial density and diversity exponentially increase (Jandhyala et al., 2015). Additionally, the gut microbiota varies greatly among individuals. Different ethnic groups in different regions possess characteristic microbial communities. The shaping of microbial communities by geographical factors is attributed to differences in dietary habits. Specifically, different dietary systems (such as the Mediterranean diet, high-fiber diet, plant-based diet, high-protein diet, ketogenic diet, and Western diet) promote the growth of certain microbial populations through their metabolites (Ross et al., 2024). For example, the Mediterranean diet promotes the proliferation of short-chain fatty acid-producing bacteria due to its richness in dietary fiber, whereas a high-protein diet may increase the abundance of protein-fermenting bacterial communities (Ross et al., 2024). The normal gut microbiota is involved in the digestion of nutrients such as sugars, lipids, and amino acids, as well as the metabolism of various drugs in the body (Armet et al., 2022; Zhang et al., 2024). Moreover, the gut microbiota contributes to immune regulation by playing a key role in sustaining normal immune signaling through its metabolites or components, like lipopolysaccharides (Lai et al., 2022). Moreover, the human microbiota is already shaped during the fetal period. Microbial metabolites present during pregnancy, along with microbial transfer during birth and immune factors passed through breastfeeding, serve as crucial sources for early-life microbiota and immune development (Koren et al., 2024). However, the “mystery” of the gut microbiota is reflected in the fact that the microbiota is not solely shaped by the previously indicated processes. Research has revealed that the dynamic interaction between the gut microbiota and the host is not limited to the local gut but widely affects physiological and pathological processes throughout the body via a multiaxis interconnected network. A classic example is represented by the gut-liver axis. Microbial-derived metabolites enter the liver through the portal vein and regulate the liver’s immune and inflammatory responses; additionally, liver-derived bile acids can reshape the gut microbiota structure via the enterohepatic circulation (Tilg et al., 2022). This dysregulation of the interaction network may lead to the occurrence of metabolic diseases and chronic liver diseases (Albillos et al., 2020). These findings suggest that there is a complex network of interactions occurring between the gut microbiota and host organs.

Recently, the gut-brain axis has garnered growing interest. Studies have shown that the gut microbiota can interact with the central nervous system (CNS) through the vagus nerve, as well as through neuroendocrine and immune pathways. Disruptions in this communication can contribute to the development of neurological disorders, including Alzheimer’s disease, Parkinson’s disease, depression, and autism (Sorboni et al., 2022; Kapoor et al., 2024). This research area is also of great interest to anesthesiologists. As a core perioperative intervention, anesthesia may be regulated by the gut microbiota in terms of its pharmacodynamics and pharmacokinetics. Therefore, this article reviews the relevant signaling mechanisms by which the gut microbiota regulates anesthesia via the gut-liver axis and gut-brain axis, summarizes the current research progress on the interaction between the gut microbiota and anesthesia, and organizes anesthesia assistance strategies based on microbial-targeted interventions.

## Signal mechanisms of the gut-brain axis in regulating anesthesia

The core aspect of research regarding the gut-brain axis is based on the elucidation of the bidirectional information transmission network between the gut microbiota and the central nervous system. This system includes four core transmission pathways: the vagus nerve pathway, the neuroendocrine pathway (the hypothalamic–pituitary–adrenal axis, HPA), the immune regulatory pathway (the cytokine network), and the microbial metabolite-mediated signaling pathway (Cryan et al., 2020).

**Figure 1:**
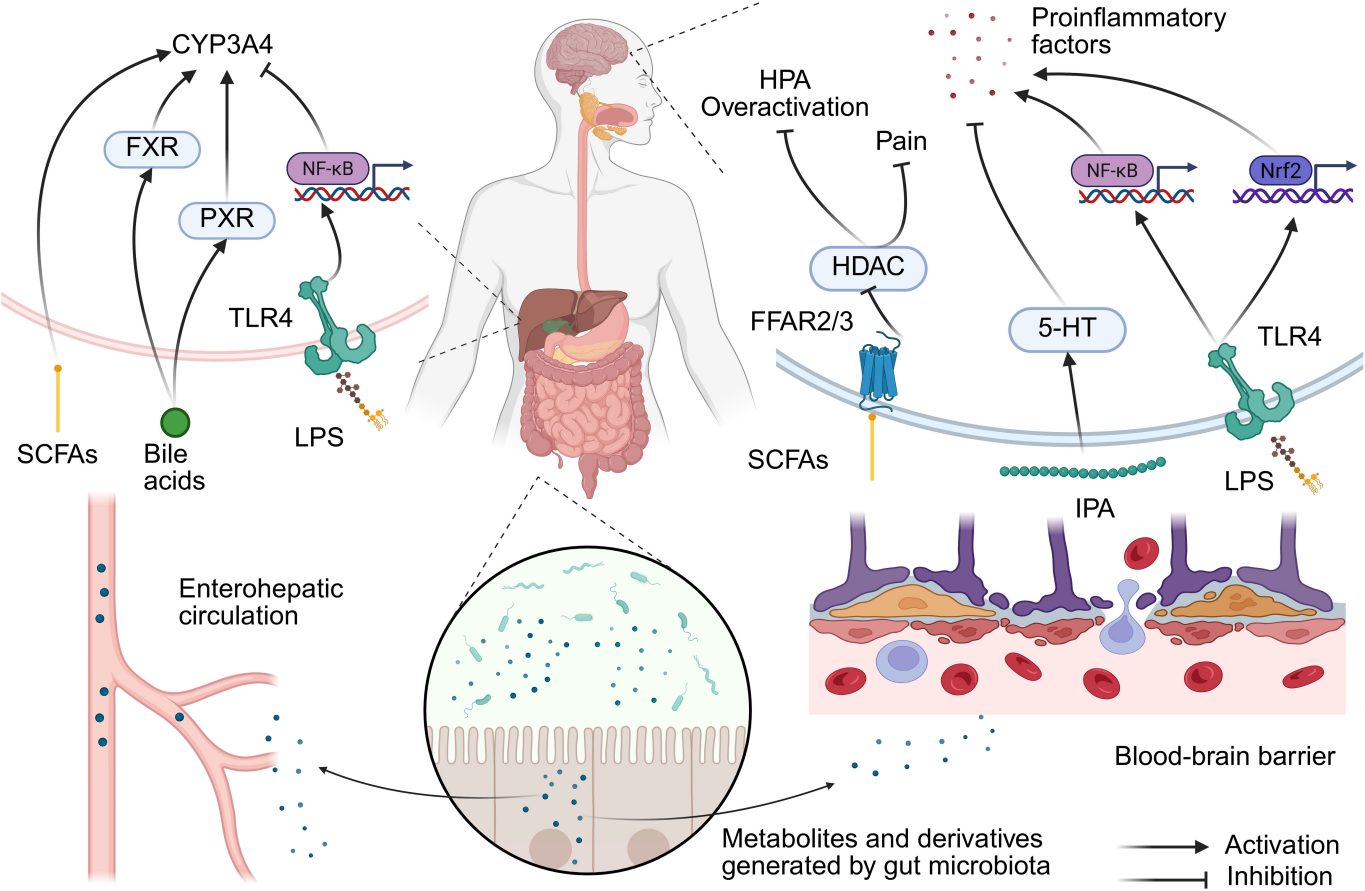
Gut microbiota regulation of the gut-liver-brain axis mechanism in anesthesia. Disrupting gut barrier integrity could promote the translocation of LPS and induces microbial dysbiosis. This triggers a dual-path response: (1) Hepatic TLR4/NF-κB activation suppresses CYP450 enzymes (e.g., CYP3A4), delaying drug clearance; (2) Systemic inflammation primes neuroinflammation via microglial NLRP3 inflammasome, exacerbating postoperative delirium. Conversely, microbial metabolites (e.g., SCFAs, IPA) exert protective effects across both axes. SCFA, short-chain fatty acid; FFAR, free fatty acid receptor; HDAC, histone deacetylase; HPA, hypothalamic–pituitary–adrenal axis; 5-HT, serotonin; IPA, indole-3-propionic acid; LPS, lipopolysaccharide; TLR4, Toll-like receptor 4; FXR, farnesol X receptor; PXR, pregnane X receptor.

Metabolites and derived products of microorganisms are the main contributors to the gut-brain axis. Short-chain fatty acids (SCFAs) are derivatives of carbohydrate breakdown induced by microorganisms and can directly act on vagus nerve afferent terminals. In mouse models, SCFAs activate vagus nerve afferent neurons by binding to free fatty acid receptors (such as FFAR2/3) or by inhibiting histone deacetylase (HDAC) activity (**Figure 1**). These signals are transmitted via the vagus nerve to the nucleus tractus solitarius (NTS) of the brainstem, which correspondingly regulates autonomic reflexes and CNS activity, thereby potentially affecting anesthesia-related sedation, pain perception, or postoperative cognitive function (Cook et al., 2021; Jameson et al., 2025). Butyric acid produced by *Roseburia intestinalis* can activate the vagus nerve via G-protein coupled receptor 41 (GPR41), thereby regulating the neural circuit from the NTS to the central amygdala, which reduces pain (Jiang et al., 2025). Interestingly, a population study (n=31) suggested that gender differences in pain thresholds among women could be partially mediated by differences in the gut microbiota-SCFAs-cortisol axis (Caputi et al., 2022). It should be pointed out that the current evidence for the mechanism of SCFAs regulating anesthesia mainly comes from animal models. Although population observational studies support their clinical relevance, the direct clinical evidence of SCFAs as anesthesia adjuncts is still limited and requires more randomized controlled trials. Microbial-derived tryptophan metabolites (such as indole-3-propionic acid) can regulate the synthesis of serotonin (5-HT) and affect the release of proinflammatory cytokines (such as IL-6 and IL-1β) in the central nervous system (Lian et al., 2021). A prospective observational cohort study revealed that baseline plasma levels of indole-3-propionic acid (IPA) were significantly negatively correlated with the onset of postoperative delirium. This relationship has also been validated in preclinical mouse models. The protective effect of IPA is partially mediated by peroxisome proliferator-activated receptor gamma coactivator 1-alpha in hippocampal interneurons (Zhou et al., 2023). Furthermore, anesthesia/surgery can lead to a decrease in gut microbiota diversity, which may disrupt the tryptophan metabolism pathway. A previous study demonstrated significant changes in the levels of tryptophan metabolites in the feces of postoperative mice, including abnormalities in key metabolites such as 5-HT precursors and kynurenine (Lian et al., 2021). In addition, beneficial gut microbiota may indirectly enhance cognitive reserve by synthesizing neuroactive compounds and enhancing brain functional connectivity. A study recruited 83 long-lived individuals over the age of 90 and 81 family members who lived with the long-lived individuals and completed fecal untargeted metabolomics and metagenomic testing. It was found that *Enterocloster asparagiformis, Hungatella hathewayi* and *Oxalobacter formigenes* were enriched in the long-lived population, and they had the ability to synthesize neuroactive substances such as gamma aminobutyric acid, glutamic acid, and guanidine (Jiao et al., 2024). This provides important clues for a better understanding of the relationship between longevity and microorganisms and their metabolites.

Lipopolysaccharide (LPS), a component of the outer membrane of gram-negative bacteria, can impair intestinal barrier function, leading to microbiota dysbiosis and allowing metabolites like endotoxins to enter the bloodstream. This, in turn, triggers a systemic inflammatory response through the “leaky gut” phenomenon. This inflammatory signal can be further transmitted to the central nervous system via increased blood-brain barrier permeability. In the hippocampus, LPS activates downstream NF-κB signaling by binding to Toll-like receptor 4 (TLR4), thereby upregulating the NOD-like receptor protein 3 (NLRP3) inflammasome, which leads to the release of inflammatory factors such as IL-1β and caspase-1. This pathway plays a central role in anesthesia-related neuropathy (Liao et al., 2022). For example, electroacupuncture treatment can improve cognitive function by inhibiting this pathway, reducing Aβ deposition and neuronal apoptosis (Liao et al., 2022). Additionally, Nrf2 is a key transcription factor for regulating antioxidant stress. LPS-induced neuroinflammation has been observed to be accompanied by a decrease in Nrf2 expression in the hippocampus, thus leading to increased oxidative damage (Kong et al., 2024). Moreover, itaconate can alleviate cognitive impairment after anesthesia and surgery by restoring Nrf2 activity, inhibiting excessive activation of microglia, and releasing proinflammatory cytokines (Kong et al., 2024). In addition, LPS-induced intestinal inflammatory signals can be transmitted via vagus nerve fibers to the central nervous system, where they activate neuroinflammatory responses in the hippocampus. Recombinant human atrial natriuretic peptide has been observed to reduce LPS-induced hippocampal inflammation and cognitive dysfunction by inhibiting vagus nerve-mediated signaling (Wu et al., 2021).

The gut microbiota forms a dynamic network with the HPA axis via metabolites, and the imbalance of this network also represents one of the pathological foundations of anesthesia-related neurological complications. SCFAs and tryptophan metabolites can directly regulate HPA axis activity and affect the secretion levels of corticosterone/cortisol. Additionally, anesthesia stress can lead to excessive activation of the HPA axis, and specific bacterial populations (such as *Lactobacilli*) can alleviate this response by inhibiting corticotropin-releasing hormone (Vodička et al., 2018; Vagnerová et al., 2019). SCFAs can penetrate the blood-brain barrier and reduce the sensitivity of the HPA axis to stress via the inhibition of HDAC, thereby improving the postanesthesia neuroinflammatory response (Rusch et al., 2023). In addition, *Akkermansia Muciniphila* has been found to regulate HPA conjugation via the peroxisome proliferators-activated receptor γ (PPAR-γ) pathway (Zhang Sh. et al., 2023).

## Anesthesia drug metabolism mediated by the gut-liver axis

The gut-liver axis represents a bidirectional crosstalk system that integrates anatomical, metabolic, and immune interactions between the gastrointestinal tract and liver. This axis regulates nutrient processing, detoxification, and immune homeostasis via mechanisms such as the enterohepatic circulation of bile acids, microbial metabolite signaling, and gut barrier integrity (Pabst et al., 2023).

The liver is the main site for the metabolism of anesthetic drugs. For example, fentanyl is mainly metabolized via cytochrome P450 enzyme (CYP3A4) activity; moreover, propofol metabolism depends on cytochrome CYP2B6, and the metabolism of rocuronium bromide is dependent on liver acetylcholinesterase. The gut microbiota can regulate liver enzyme activity through the gut-liver axis. SCFAs (such as acetic acid) produced by the gut microbiota can enter the liver via the blood circulation as precursors of acetyl-CoA, thereby affecting histone acetylation and regulating CYP3A4 expression (Liu et al., 2024). Dysbiosis of the gut microbiota can lead to LPS translocation to the liver, along with activating inflammatory pathways such as TLR4/NF-κB and inhibiting CYP3A4 activity (Liu et al., 2024). Moreover, the gut microbiota has been shown to affect the activity of farnesol X receptor (FXR) and pregnane X receptor (PXR) by regulating bile acid metabolism. These nuclear receptors can directly regulate the expression of CYP3A4 and associated transporters, thereby altering the metabolic rate and clearance efficiency of anesthetic drugs (Luo et al., 2022; Cao et al., 2024). The structure and concentration of bile acids can also directly influence the effects of certain anesthetic drugs. For example, cholic acid and its derivatives can prolong the local anesthesia time of lidocaine, and its mechanism may involve alterations of cell membrane permeability or drug distribution (Posa et al., 2007). In models of bile stasis (such as bile duct ligation), the concentration of hepatic microsomal cytochrome P450 has been observed to be significantly reduced, thus leading to a decrease in the oxidative metabolism ability of anesthetic drugs (Schacter et al., 1983). Moreover, pentobarbital may further affect bile flow and composition by altering bile osmotic activity or the excretion of metabolites in this state (Kuipers et al., 1985). In addition, anesthetic drugs can also affect the process of bile acid metabolism. Specifically, propofol can inhibit the activity of CYP27A1 (a key enzyme in bile acid synthesis) by binding to this enzyme, thereby leading to abnormal bile acid metabolism and inducing bile stasis and dysbiosis of the microbiota (Claesen et al., 2020). Therefore, in patients experiencing bile stasis or liver dysfunction, special attention should be given to the selection and dosage adjustments of anesthetic drugs.

It is worth noting that the impact of gut microbiota on the metabolism of anesthetic drugs varies widely among different populations. Previous studies have shown that the gut microbiota of individuals of different ages, body masses, sexes and nationalities can be roughly divided into three types, namely, *Bacteroides* (more represented in enterotype 1), *Prevotella* (more abundant in enterotype 2), and *Ruminococcus* (prevalent in enterotype 3) (Wu et al., 2011; Shanahan et al., 2017). However, the composition of gut microbiota varies among different geographical populations, and this is mostly attributed to differences in dietary habits. For example, the typical westernized dietary pattern is a high-sugar, high-fat, high-protein and low-fiber diet, which leads to decreased α-diversity, increased β-diversity and decreased abundance or even the extinction of *Prevotella* and *Treponema* species54. A high fiber/vegetarian diet is associated with an increase in gut microbiota diversity, where indigestible polysaccharides can be fermented into SCFAs by the gut microbiota. This dietary pattern can improve patients’ response to anesthetics (such as reducing postoperative pain) (Minerbi and Shen, 2022). The mediterranean diet emphasizes eating more fruits, vegetables, legumes, and grains, and eating less meat. This diet pattern can improve the high risk factors of anesthesia such as obesity, diabetes and insulin resistance (Ruiz et al., 2019). With the adherence to mediterranean diet, the potential pro-inflammatory *Ruminococcus gnavus* has also decreased, indicating that mediterranean diet has anti-inflammatory effects and may optimize the physiological response of patients to anesthetic drugs and surgical trauma (Ghosh et al., 2020).

## Effects of anesthesia on the gut microbiota

### The shaping of the gut microbiota by anesthetic drugs

Anesthetics can either directly or indirectly (via changes in factors such as pH, oxygen partial pressure, and mucus secretion) disrupt the gut microbiota, thus leading to a decrease in microbiota diversity, the proliferation of proinflammatory microbiota, and the development of metabolic disorders, which can correspondingly affect central nervous system function via immune, neurological, and endocrine pathways. Animal experiments have demonstrated that propofol exerts a certain antibacterial effect, and the specific mechanism may involve the inhibition of microbial growth by disrupting the integrity of the cell membrane. Moreover, the results of these experiments demonstrated that, at three hours after rats were injected with propofol, the abundances of *Prevotella* and *Lactobacillus* at the genus level decreased, with these bacteria exhibiting a recovery trend of increased abundances on the 14th day (Minerbi and Shen, 2022). The impact of inhaled anesthetics on the gut microbiota is relatively significant and may directly or indirectly cause alterations in the composition of the gut microbiota, such as by reducing the abundance of *Lactobacilli*, thereby affecting vagal nerve transmission signals (Liufu et al., 2020). This difference suggests that clinical interventions need to consider the effects of anesthesia duration, drug type, and host factors (such as age and underlying diseases) on the dynamics of microbiota recovery. Previous studies have demonstrated that after the administrations of multiple sevoflurane anesthetics, the abundance and alpha diversity (including measurements of the Shannon index) of the gut microbiota significantly decreased. Specifically, the abundance of beneficial bacteria like *Akkermansia* was significantly reduced, while the relative abundance of certain potentially pathogenic bacteria, such as *Streptococcus*, increased (Han et al., 2021; Han et al., 2024). This change also showed a recovery trend at 14 days (Han et al., 2021). Age also affects the sensitivity of the microbiota to sevoflurane. For example, repeated exposure to sevoflurane anesthesia during the neonatal period can significantly alter the gut microbiota of mice (including abnormal abundances of *Streptococci* and *Spirochetes*, among other bacteria) and is associated with cognitive impairment (Liu et al., 2021). The gut microbiota of the elderly population is inherently fragile, and anesthesia and surgery further exacerbate the imbalance of the microbiota, leading to an increased risk of postoperative delirium (Zhang Y. et al., 2023). Mechanistically, sevoflurane alters gut microbiota function by reducing the synthesis of secondary bile acids, such as deoxycholic acid (Zhao et al., 2024). Moreover, exposure to sevoflurane may lead to an increase in the microbial derivative trimethylamine oxide (TMAO), which in turn activates NLRP3 in microglia, stimulates the release of proinflammatory cytokines (such as IL-1β, IL-6, and TNF-α), and induces neuroinflammation (Zhao et al., 2019; Huang et al., 2021; Han et al., 2024). Isoflurane, as another commonly used inhaled anesthetic, can lead to a decrease in gut microbiota diversity. After 4 hours of exposure to isoflurane, the abundance of *Firmicutes*, *Clostridia*, *Clostridiales*, and *Lachnospiraceae* in the fecal microbiome of rats significantly increased, while the abundance of *Bacteroidetes*, *Actinobacteria*, *Bacteroides*, and *Bacteroidaceae* significantly decreased (Wang et al., 2019). Isoflurane also contributes to the accumulation of inflammatory cytokines (such as IL-1β and IL-6) in the central nervous system, which is closely associated with postoperative delirium symptoms, including behavioral and cognitive impairments (Jiang et al., 2019).

Opioid receptors (such as μ receptors) are widely expressed in intestinal epithelial cells and immune cells. Therefore, opioid drugs such as morphine can directly inhibit the secretion of antimicrobial peptides, alter intestinal pH and redox status, and selectively promote the proliferation of pathogenic bacteria while inhibiting beneficial bacteria (Thomas et al., 2022; Kolli et al., 2023). Numerous studies on rodents have shown that opioid drugs can cause rapid and significant dysbiosis of the microbiota. After discontinuation of medication, some microbial parameters may recover within several weeks or even longer, but complete recovery to baseline status is rare (Thomas et al., 2022). Mechanistically, opioid drugs impair intestinal barrier integrity by inhibiting intestinal peristalsis, reducing mucus secretion, and disrupting tight junction proteins (such as ZO-1 and occludin), thereby promoting the translocation of bacteria and their metabolites (including the entry of LPS into the bloodstream) (Zhang and Roy, 2021). Moreover, opioid drugs inhibit the production of SCFAs (such as butyrate and propionate) and suppress the signal of the FXR, thereby leading to the accumulation of primary bile acids and a decrease in secondary bile acids (Gicquelais et al., 2020). The accumulation of these harmful metabolites further exacerbates neuroinflammation, forming a vicious cycle. As a result, morphine and fentanyl can significantly reduce the alpha diversity of the gut microbiota (including reductions in the Shannon index and observed operational taxonomic units), which is a phenomenon observed in both human patients and animal models (Gicquelais et al., 2020). In terms of changes in the abundance of specific bacterial genera, *Roseburia*, which produces butyrate, decreased, and *Bilophila*, which participates in bile acid metabolism, decreased. And *Bacteroides* may become the dominant bacterial group, associated with various inflammatory responses (Gicquelais et al., 2020) (**Table 1**).

**Table 1:** The impact of anesthetic drugs on the gut microbiota.

Anesthetic drug	Alteration in gut microbiota	Possible mechanisms	Evidence level	References
Propofol	*Prevotella* and *Lactobacillus↓*	Destruction of cell membrane integrity	Animal study	(Claesen et al., 2020)
Sevoflurane	*Akkermansia↓*; *Streptococcus↑*	Reduction of secondary bile acid synthesis, promotion of the release of pro-inflammatory cytokines by TMAO-NLRP3	Animal study	(Wu et al., 2011; Liufu et al., 2020; Minerbi and Shen, 2022; Han et al., 2024)
Isoflurane	*Firmicutes*, *Clostridia*, *Clostridiales*, and *Lachnospiraceae↑*; *Bacteroidetes, Actinobacteria, Bacteroides*, and *Bacteroidaceae↓*	Accumulation of IL-6 and IL-1β in CNS	Animal study	(Liu et al., 2021; Zhang Y. et al., 2023)
Opioid drugs	*Roseburia*↓, *Bilophila*↓; *Bacteroides*↑	Disruption of gut barrier and reduction in the generation of beneficial microbial derivatives such as SCFAs	Human & Animal study	(Wang et al., 2019; Huang et al., 2021)

↑, upregulation; ↓, downregulation.

### Potential risks of long-term anesthesia exposure

Intensive care unit (ICU) patients often experience dysbiosis due to long-term sedation therapy (such as continuous infusion of midazolam), which may exacerbate increased intestinal permeability and systemic inflammatory response. A prospective cohort study of 61 ICU patients found that the abundance of *Bifidobacterium* genus was significantly higher in surviving patients compared to those who died during hospitalization (Kang et al., 2017). Another prospective study included 577 premature infants admitted to the ICU, collecting gut microbiota and conducting cluster analysis through 16S ribosomal RNA gene sequencing. The results showed that Cluster 4 (driven by *Enterococcus*) and Cluster 5 (driven by *Staphylococcus*) were associated with lower average gestational age, while Cluster 3 (driven by *Escherichia*/*Shigella*) was associated with higher average gestational age (Rozé et al., 2020). These findings suggest that certain cluster analysis of microbial communities may be noninvasive biomarkers. Additionally, long-term sedation may lead to decreased gastrointestinal motility and insufficient intake of enteral nutrition. Low enteral nutrition is significantly correlated with high-risk microbial clustering (such as Cluster 5), thereby indicating that inadequate nutritional support may exacerbate microbial dysbiosis (Rozé et al., 2020). In addition, ICU patients commonly use broad-spectrum antibiotics and experience prolonged courses of treatment, which further exacerbates microbiota disorders (Iapichino et al., 2008). In summary, critically ill ICU patients exhibit significant dysbiosis of the gut microbiota. However, the potential role of microbial-targeted therapy measures has not yet been determined.

### The gut microbiota and postoperative pain

The relationship between the gut microbiota and pain has been a research hotspot in the biomedical field in recent years and involves the investigation of multiple types of pain (such as visceral pain, neuropathic pain, inflammatory pain, headache, and cancer pain, among other types) and complex regulatory mechanisms (Minerbi and Shen, 2022). With respect to postoperative pain, the gut microbiota can participate in the occurrence of pain by affecting signaling of the vagus nerve and the release of inflammatory factors (Guo et al., 2019; Lee et al., 2023). In addition, preoperative antibiotic-induced dysbiosis can increase visceral pain sensitivity, whereas the restoration of microbial balance can reverse pain (Lucarini et al., 2022). A previous study revealed that preoperative microbiota characteristics can predict the risk of chronic postoperative pain. The decrease in the abundance of certain bacteria in breast cancer patients before surgery may be related to the increase in pain sensitivity (Yao et al., 2022). Following fecal microbiota transplantation, recipient mice with a “chronic postoperative pain microbiota” showed a significant increase in mechanical hyperalgesia and a decrease in the expression of Ppar-γ and arginase-1 in the spinal cord (Yao et al., 2022). Drugs such as somatostatin may reduce the risk of pancreatic fistula related pain by regulating postoperative microbiota composition (such as via reductions in *Enterobacteriaceae*) (Li et al., 2020). In summary, the causal mechanism of the relationship between the microbiota and postoperative pain, as well as individualized intervention plans, still need to be further explored, especially regarding the validation of different surgical types and patient populations (Guo et al., 2019; Morreale et al., 2022).

## Anesthesia intervention strategy based on gut microbiota assistance

### Microbial metabolite regulation

SCFAs: The first potential application of SCFAs involves anesthesia assistance, as certain SCFAs (or their metabolites) may produce anesthetic-like effects by enhancing the inhibitory neurotransmitter system. For example, acetic acid can enhance the anesthetic effect of sevoflurane and reduce its required concentration, which may be mediated by adenosine A1 receptors (Carmichael et al., 1991). Moreover, isovaleric acid exhibits direct anesthetic activity in tadpole models. Although propionic acid and methyl malonic acid do not exhibit separate anesthetic effects, they can significantly reduce the half effective concentration of isoflurane, thereby suggesting that they may be used as anesthetic adjuvants (Weng et al., 2009). Additionally, β-hydroxybutyric acid (a ketone body possessing a similar structure to the structures of SCFAs) produces anesthetic effects by enhancing GABAA receptor function, which is similar to the state of consciousness inhibition observed in ketotic patient (Yang et al., 2007). The second potential application involves assistance in postoperative recovery. Research has shown that supplementation with SCFAs (such as the regulation of the microbiota via the diet or electroacupuncture) may reduce postanesthesia neuroinflammation and cognitive impairment, especially in elderly or high-risk patients (Xu et al., 2021; Ke et al., 2022).

Other metabolic pathways: The associations between bile acid levels and anesthesia treatment mainly involve drug metabolism, enhanced treatment efficacy, and alternative treatment strategies. For patients with bile acid secretion disorders (such as progressive familial intrahepatic cholestasis) or liver injury, general anesthesia may exacerbate metabolic burdens, and the careful selection of anesthesia regimens is necessary (Clifton et al., 2011). Cholic acid and its derivatives have been found to enhance the local anesthetic effect of lidocaine (Posa et al., 2007). In addition, for high-risk patients who cannot tolerate anesthesia or surgery, piezoelectric lithotripsy combined with oral bile acids (such as ursodeoxycholic acid) is a safe and effective option. Research has shown that 69% of patients with single stones can achieve complete gallbladder clearance after lithotripsy without anesthesia (Pelletier et al., 1991). The supplementation of tryptophan metabolites can also represent a target for anesthesia intervention. For example, electroacupuncture treatment restores gut-brain axis function and reduces postoperative cognitive impairment and neuroinflammation by increasing the number of IPA-producing bacteria and their metabolites (Tang et al., 2024). Furthermore, the direct supplementation of IPA or the inhibition of key enzymes in the canine urinary ammonium pathway (such as IDO1) may become a new strategy for preventing and treating postoperative delirium (Zhou et al., 2023).

### Antibiotics and probiotics

Antibiotics and probiotics are both ways that can directly alter the composition of the gut microbiota. Although antibiotics aim to prevent infections, they may increase the risk of complications. Prophylactic antibiotics during the perioperative period are one of the main factors that induce allergic reactions during anesthesia (Yasuda et al., 2021). Some antibiotics also have neurotoxic potential. Beta-lactam antibiotics (especially cephalosporins and carbapenems) are strongly associated with seizures and encephalopathy. Fluoroquinolones and macrolides can cause psychosis, insomnia, and neuropathy (Radkowski et al., 2025). This neurotoxicity, combined with the neuroprotective effect of anesthetic drugs themselves, may exacerbate the neurological damage in patients. Therefore, clinical decision-making needs to balance allergy history, surgical type, and anesthesia duration, and pay attention to the compatibility of medication timing with guidelines.

The preoperative or postoperative use of probiotics may alleviate anesthesia and surgery-related inflammatory reactions by regulating the balance of the gut microbiota. For example, in cardiac surgery, the combination of selective gastrointestinal decontamination, probiotics, and montmorillonite pretreatment can significantly reduce postoperative endotoxin and IL-6 levels and alleviate systemic inflammatory responses (Liu et al., 2018). In addition, probiotics may reduce the risks of postoperative infections and neurocognitive complications by maintaining the integrity of the intestinal barrier, thereby reducing the overgrowth of pathogenic bacteria and the release of metabolites such as endotoxins (Zanza et al., 2022). Adding specific beneficial bacteria (such as *Lactobacilli* and *Bifidobacteria*) has been found to improve cognitive function by regulating neuroinflammation and oxidative stress response (Liu et al., 2022; Tian et al., 2024). Currently, the auxiliary effect of probiotics on anesthesia is still controversial in human research. Different strains, doses, and timings of administration can also affect treatment efficacy. Therefore, it is necessary to combine individualized strategies (such as the combination of prebiotics and probiotics) to achieve optimal results.

### Fecal microbiota transplantation

FMT is mainly used to treat recurrent *Clostridium difficile* infections by transplanting functional microbiota from healthy donor feces into the patient’s gut to restore gut microbiota balance (Khoruts et al., 2021). Currently, only preliminary studies have been performed on FMT-assisted anesthesia therapy. Animal studies have demonstrated that the correction of anesthesia-induced microbiota abnormalities via FMT may improve cognitive function. After the transplantation of healthy microbiota, cognitive impairment in young mice was observed to be alleviated, thereby suggesting that FMT may be a treatment for anesthesia-related neurotoxicity (Liu et al., 2021). The long-term use of antibiotics leading to gut microbiota dysbiosis (such as antibiotic-associated diarrhea) is a common problem observed in ICU patients. A prospective, multicenter, randomized controlled trial (NCT05430269) including 36 ICU patients with antibiotic-associated diarrhea revealed that FMT can accelerate diarrhea relief by restoring gut microbiota diversity (Cibulkova et al., 2024). However, patients under anesthesia or sedation should be given increased attention regarding operational safety when undergoing FMT. At the technical level, the technique involving the insertion of the middle section of the naso-intestinal tube has been proven to be safe and reliable. In addition, when ICU patients exhibit weakened immune function, FMT may cause bacterial translocation or systemic inflammatory reactions. Moreover, it is necessary to strictly screen donor feces and perform long-term cryopreservation (3–12 months) to reduce the risk of infection (Cibulkova et al., 2024). In conclusion, the efficacy and safety of FMT in anesthesia-exposed populations need to be systematically evaluated via more clinical trials, especially in terms of its preventive or therapeutic value for patients with cognitive impairments after the administration of multiple anesthetics. Moreover, the optimization of the implementation standards of FMT during the perioperative period, including donor selection, bacterial solution preparation methods, and administration timing, is necessary.

### Difficulties and directions in clinical translation

Although significant progress has been made in recent years in the study of the mechanism of interaction between gut microbiota and anesthesia, and potential pathways for regulating anesthesia efficacy and postoperative complications through the gut-brain-liver axis, as well as auxiliary strategies based on microbiota intervention, there is still a significant gap between current research and clinical application. Firstly, the depth of clinical evidence is insufficient. Most of the evidence for core signaling mechanisms comes from rodent models. There are differences in human physiology, immune system, and microbiota composition compared to animal models, and the exact pathways, strength of effects, and targets of these mechanisms in the human body still need to be directly validated in clinical populations. Existing human studies are mostly observational studies, making it difficult to establish whether changes in microbiota are a cause or a result of anesthesia/surgical complications. In addition, the significant impact of host factors (age, genetic background, dietary habits, underlying diseases, and concomitant medications) on microbiota anesthesia interactions has not been systematically quantified and integrated in clinical studies. This makes it difficult to predict an individual’s response to anesthesia and sensitivity to microbiota intervention. Secondly, the breadth of clinical evidence is insufficient. At present, the number of clinical studies and the sample size is small, the observation period is short, and they are mostly concentrated in specific populations (such as ICU patients and specific surgical types), lacking universal conclusions. The lack of standardization in intervention plans makes it difficult to compare and generalize research results. In addition, we know very little about the potential long-term effects of microbiota regulation on anesthesia, such as its impact on immune homeostasis and metabolism.

**Figure 2:**
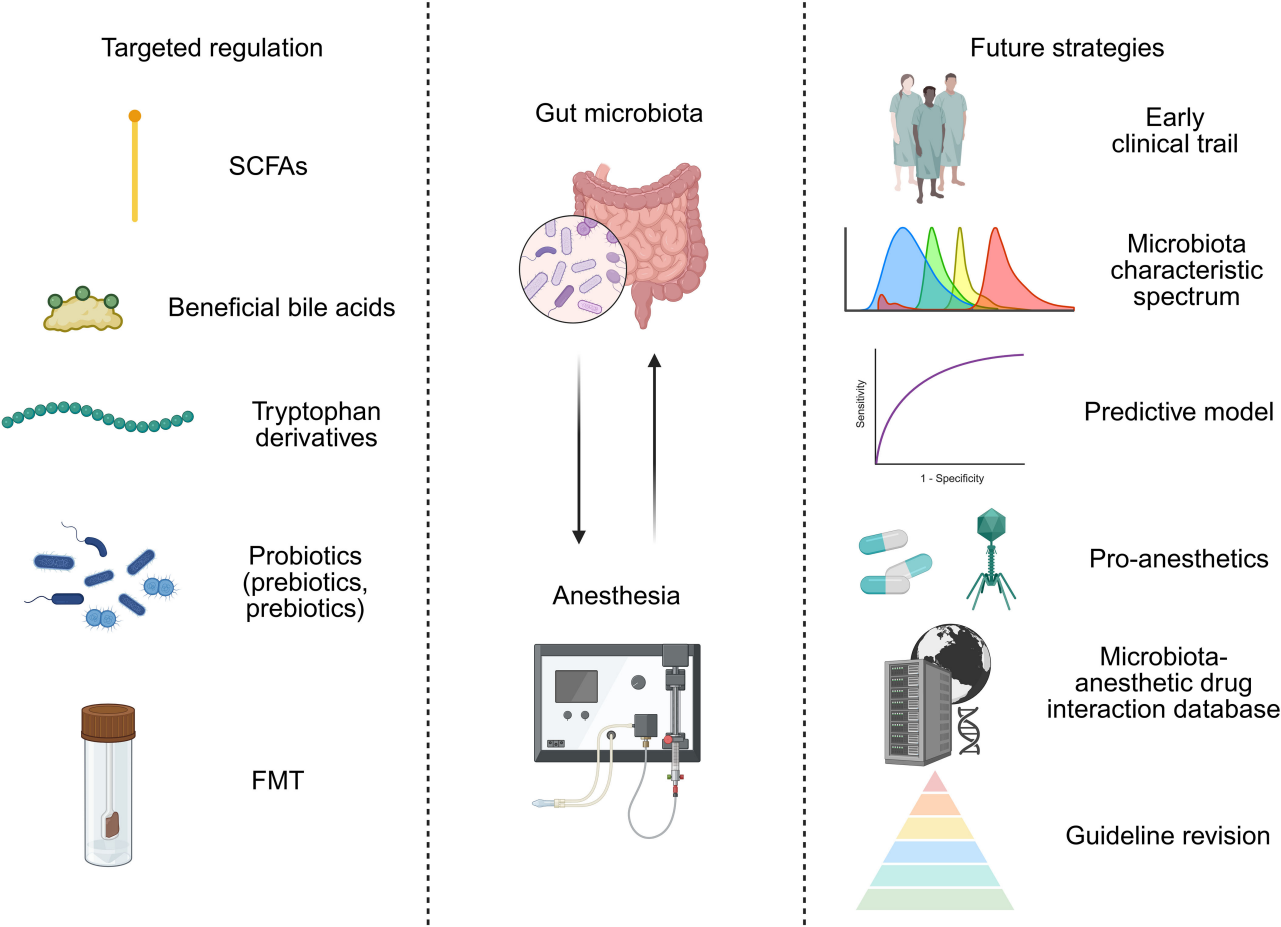
Targeted modulation of the gut microbiota for precision anesthesia: approaches and future strategies.

Therefore, in order to overcome these clinical translation bottlenecks, possible directions include: 1) designing early clinical trials to evaluate the pharmacokinetics, safety, and preliminary efficacy of key microbial metabolites (such as specific SCFAs, IPA) in healthy volunteers or specific patient populations (such as reducing dosage as an anesthetic adjuvant). Combined with biological sample analysis, verify whether it reaches the target tissue and activates predetermined pathways (such as detecting receptor occupancy and downstream inflammatory marker changes). 2) Conduct a large-scale, prospective perioperative cohort study, systematically collect preoperative fecal microbiota, blood metabolome, and host genome data, and record anesthesia regimens, surgical types, and postoperative complications in detail. Integrate multidimensional data (including age, BMI, preoperative microbiota alpha diversity, abundance of key bacterial genera, baseline serum SCFAs, and host drug metabolism gene polymorphism such as CYP2B6) through machine learning (such as integrating XGBoost and random forest algorithm), screen microbiota characteristic spectra highly correlated with anesthesia sensitivity and postoperative cognitive impairment, and establish a clinically deployable predictive model (Lu et al., 2025). 3) Conduct multicenter, large sample, placebo-controlled randomized controlled studies targeting specific high-risk populations, such as elderly patients and patients undergoing major surgery. Clarify the optimal strain combination, dosage, timing of administration, and course of treatment. 4) Developing pro-anesthetics that can accurately determine their location in the intestine or across the blood-brain barrier, and promoting their early clinical evaluation, with the aim of improving intervention efficiency and reducing systemic side effects. For instance, construct a chitosan sodium alginate nanocarrier system loaded with butyric acid prodrugs (such as triglycerides) to achieve targeted regulation of the gut-brain axis; alternatively, CRISPR-Cas9 engineered bacteriophages can be designed to precisely inhibit specific metabolic pathways (such as butyryl CoA dehydrogenase gene) of perioperative pathogenic bacteria and reduce the production of liver toxicity intermediates related to propofol metabolism (Chen et al., 2023; Fan et al., 2024). 5) Build a “microbiota-anesthetic drug interaction database”. 6) Based on accumulated evidence, develop consensus or preliminary guidelines on the use of probiotics, prebiotics, or specific dietary interventions during the perioperative period (**Figure 2**). Through this step-by-step effort, there is hope for gut microbiota and anesthesia to move from descriptive association studies to mechanistic causal verification, thereby assisting clinical treatment decisions.

## Conclusions and perspectives

In recent years, the interaction between the gut microbiota and anesthesia has gradually become a popular topic in interdisciplinary research. Via multidimensional interaction mechanisms such as the gut-liver axis and gut-brain axis, the gut microbiota not only participates in the metabolic regulation of anesthetic drugs but also may directly affect the functional status of the central nervous system through metabolites (such as SCFAs and neurotransmitter precursors), thereby regulating the depth of anesthesia and the postoperative recovery process. Research has shown that specific strains and their metabolites can directly or indirectly affect the clearance rate and toxicity threshold of anesthetic drugs by regulating the activity of liver drug-metabolizing enzymes. In addition, the activation of gut-brain axis signals (such as the vagus nerve pathway and inflammatory cytokine release) has been demonstrated to be closely related to the occurrence of postoperative delirium (Zhang Y. et al., 2023). However, the anesthesia process itself also exerts a dynamic reshaping effect on the gut microbiota. Perioperative stress, antibiotic exposure, and anesthetic drugs may significantly alter the composition and function of the gut microbiota. This bidirectional interaction suggests that anesthesia management needs to balance the maintenance of microbial homeostasis to avoid complications caused by imbalanced microbiota. Studies have preliminarily revealed the potential value of microbial interventions, including probiotics (such as *Bifidobacterium*), synbiotics, and FMT, which have demonstrated potential in animal models for alleviating anesthesia-related intestinal dysfunction and shortening recovery times. Specific probiotic strains can repair the intestinal mucosal barrier by secreting butyric acid, reducing the entry of endotoxins into the bloodstream, and consequently decreasing the postoperative systemic inflammatory response.

With the thorough integration of microbiology and anesthesia pharmacology, the future clinical application prospects regarding the microbiota-anesthetic relationship are broad. For example, preoperative dietary interventions or prebiotic pretreatments can reshape the patient’s gut microbiota and improve anesthesia drug tolerance. During surgery, bacteriophages can be used to precisely inhibit the metabolic pathways of pathogenic bacteria, thereby reducing the hepatotoxicity of anesthetic drugs. Additionally, after surgery, an early warning system based on microbial biomarkers could be used in conjunction with FMT to reduce the risks of complications such as delirium and infection. Explorations in this field may transition perioperative medicine into a new era of ‘microbiota-targeted therapy’. However, the progression from the laboratory setting to the clinical setting still requires interdisciplinary collaboration and technological breakthroughs. In the future, it will be necessary to reveal the tripartite interaction law between microorganisms, hosts, and drugs, as well as promote the transformation of the “precision microbial assisted anesthesia” mode and ultimately improve surgical safety and long-term patient prognosis. These research directions can lead to a truly innovative path for individualized anesthesia management and postoperative rehabilitation.
